# Sustained improvement in caregiver-reported infant demographic collection: a pilot towards equitable outcomes in the neonatal intensive care unit

**DOI:** 10.1038/s41372-026-02676-z

**Published:** 2026-05-18

**Authors:** Megan Barcroft, Bret Davis, Roopali Bapat, Tara Cosgrove, Stephanie Napolitano

**Affiliations:** 1 Division of Neonatology, Nationwide Children’s Hospital, Columbus, OH USA; 2 Center for Clinical Excellence, Nationwide Children’s Hospital, Columbus, OH USA; 3 Office of Health Equity, Nationwide Children’s Hospital, Columbus, OH USA

**Keywords:** Paediatrics, Health services

## Abstract

**Background:**

Infant mortality disparities, with prematurity among the leading causes of death, make it critical for neonatal intensive care units (NICUs) to assess internal demographic and outcomes data. Assessment of a delivery hospital NICU within a large children’s hospital system revealed “Unavailable” infant race among the top demographic categories.

**Objective:**

To decrease the rate of “Unavailable” infant race in the electronic health record by 10% in a level III NICU by December 2023 and sustain for 1-year.

**Design/methods:**

We developed a multidisciplinary team, completed Gemba walks, conducted Plan-do-study-act (PDSA) cycles, and analyzed outcomes in process control charts.

**Results:**

“Unavailable” infant race decreased from 97% to 49% and was sustained. Modifying hospital registration processes, updating caregiver questionnaires, engaging and educating staff contributed to success and sustainment.

**Conclusions:**

We reduced “Unavailable” data by 48% with 6-months sustainment prior to project expansion. Emulating this process can improve demographic data accuracy nationally.

## Introduction

Infant mortality disparities persist nationally, with complex historical roots of systemic racism leading to inequitable preterm birth rates among birthing people [1]. In Ohio, the mortality of non-Hispanic Black infants remains 2-3x higher than that of White infants over the last decade [2]. In response, the Governor designated *Maternal and Infant Health* as a priority health outcome in the 2020 State Health Improvement Plan [3]. The action-oriented strategy started with identification of priority populations, followed by allocation of tailored resources. Prematurity was identified as the leading cause of statewide infant death, with non-Hispanic Black populations and residents of urban counties as the highest priorities. Community collaborations and government funding fueled the formation of the Ohio Equity Institute, where utilization of population data focused resource allocation to the counties with the largest disparities [3].

With prematurity as the leading cause of statewide infant death, neonatal intensive care units (NICUs) are called to lead their hospitals’ efforts toward health justice by identifying and correcting disparities in care and outcomes. Healthcare disparities are differences observed in the way groups of people access, experience, and receive healthcare [4]. Healthcare disparities can occur due to missed care or care that causes harm, impacted by social, economic, environmental, and other factors [4, 5]. Studies investigating the relationship between NICU patient demographics and socioeconomic status, outcome metrics, and quality care indicators have been mixed, highlighting the importance of regional assessment of quality and outcomes [6–8].

To perform intentional and thorough analyses aimed at identifying potential differing outcomes, it is essential to first obtain complete and accurate sociodemographic patient information [9]. Longstanding concerns regarding the accuracy of electronic health record (EHR) patient demographic documentation have been raised in adult healthcare, with evidence of largescale discrepancies resulting in a persistent call-to-action for improvement in collection processes [10–13]. A survey of 93 pediatric hospitals showed extreme variability among collection practices of patient race, ethnicity, and language data, with only 31% of hospitals collecting parental demographics, a wide variety in demographic category use, a low rate of written standardized policies, and a subpar 70% of hospitals providing formal staff training [14]. Pediatric emergency departments and intensive care units have been shown to be at particular risk for inaccurate documentation given the high acuity and complex admission processes [15, 16]. Most recently, a cross-sectional study of the 3 largest pediatric hospitals in Michigan further elevated validity concerns with a patient race mismatch rate between parental reporting and EHR documentation of 41–78%, with up to 10% missing data [17].

In efforts to assess our performance, we conducted an internal review of all infant discharges in 2022 within our 10-unit, 4-hospital system Neonatal Network. The analysis revealed the second most common infant race documented at the time of discharge as “Unavailable,” with one unit having a rate as high as 97%. Site-specific analysis identified heterogeneous practices, with no consistent intake process throughout the neonatal network. With this objective, we conducted a pilot, single-site quality improvement (QI) study in our NICU where the rate of “Unavailable” infant race data was highest. The specific aim was to decrease the documentation of infant race as “Unavailable” at the time of discharge or transfer from 97% to 87% in a Level III NICU by December 1, 2023, and sustain for one year.

## Methods

### Context

The pilot site for this improvement work is a level III, 49-bed, delivery unit, with an average of 915 yearly admissions and part of a larger Neonatal Network. In collaboration with Maternal-Fetal Medicine colleagues, the unit serves a broad, high-risk delivery population, including those born with surgical complexities or prematurely as early as 22 weeks. Approximately half of the population served are publicly insured with 5 percent of families preferring non-English languages. While Nationwide Children’s Hospital (NCH) owns and operates this unit, the physical location is housed inside an adult hospital, “off-site” from the main NCH campus. Both the pilot NICU and main NCH campus are located in urban areas in a county designated by the Ohio Equity Institute as high priority for infant health initiatives, further emphasizing the importance of accurate patient demographic documentation [3].

**Fig. 1:**
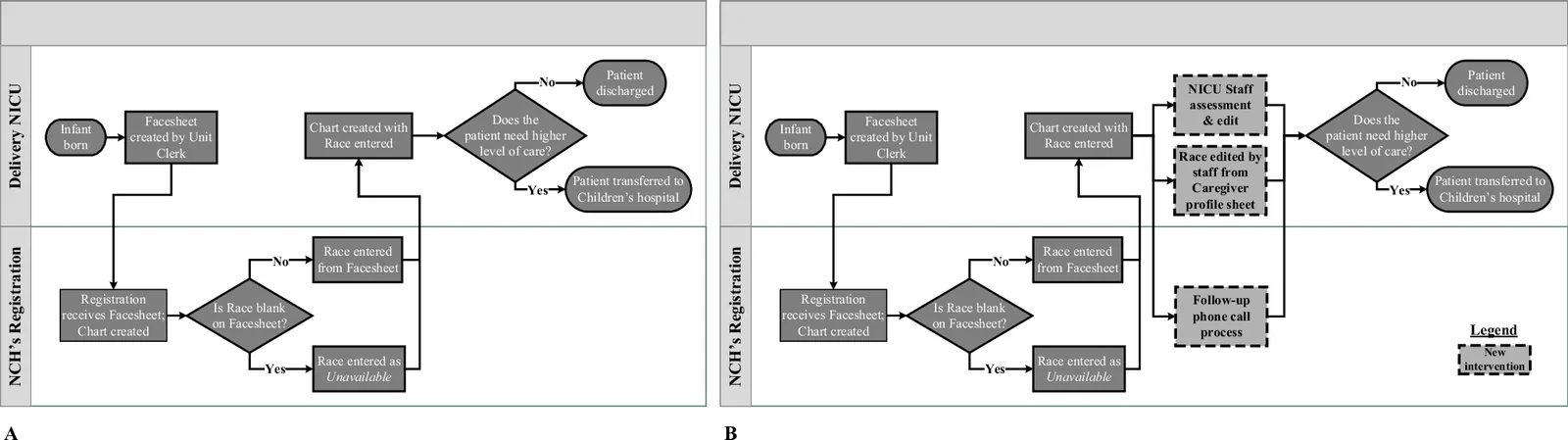
Swimlane Process Map. **A** Baseline process, **B** Current process.

Scouring EHR data (Epic Systems Corporation 2023, Verona, Wisconsin) was paramount to this work. The initial assessment of NICU admissions from January to December 2022 through Epic SlicerDicer revealed a high number of “Unavailable” demographics. We performed Gemba walks, a fundamental Lean Six Sigma tool, where members of the team travel to the place where the actual work is done to improve process understanding [18, 19]. Following multiple Gemba walks across the network and hospital enterprise, we mapped the process of infant demographic documentation (Fig. 1). At the time of admission, most families receive a nurse-led, informal orientation to the unit, followed by a universal social work consultation and psychosocial assessment, occurring ideally within the first 24 h of admission. Parents are also asked to complete a caregiver profile questionnaire, which gathers information on items such as preferences for learning methods, recent travel, and infant feeding plans. These processes introduce families to the NICU environment; however, most parents do not interact with registration staff nor complete an institutional registration process, leaving gaps in our ability to fully understand and support the infant and family. For a more rigorous understanding of how the demographic documentation occurred, we automated a weekly EHR report with our Data Analytics team to collect staff names and time stamps corresponding to any edits made to demographics.

**Fig. 2:**
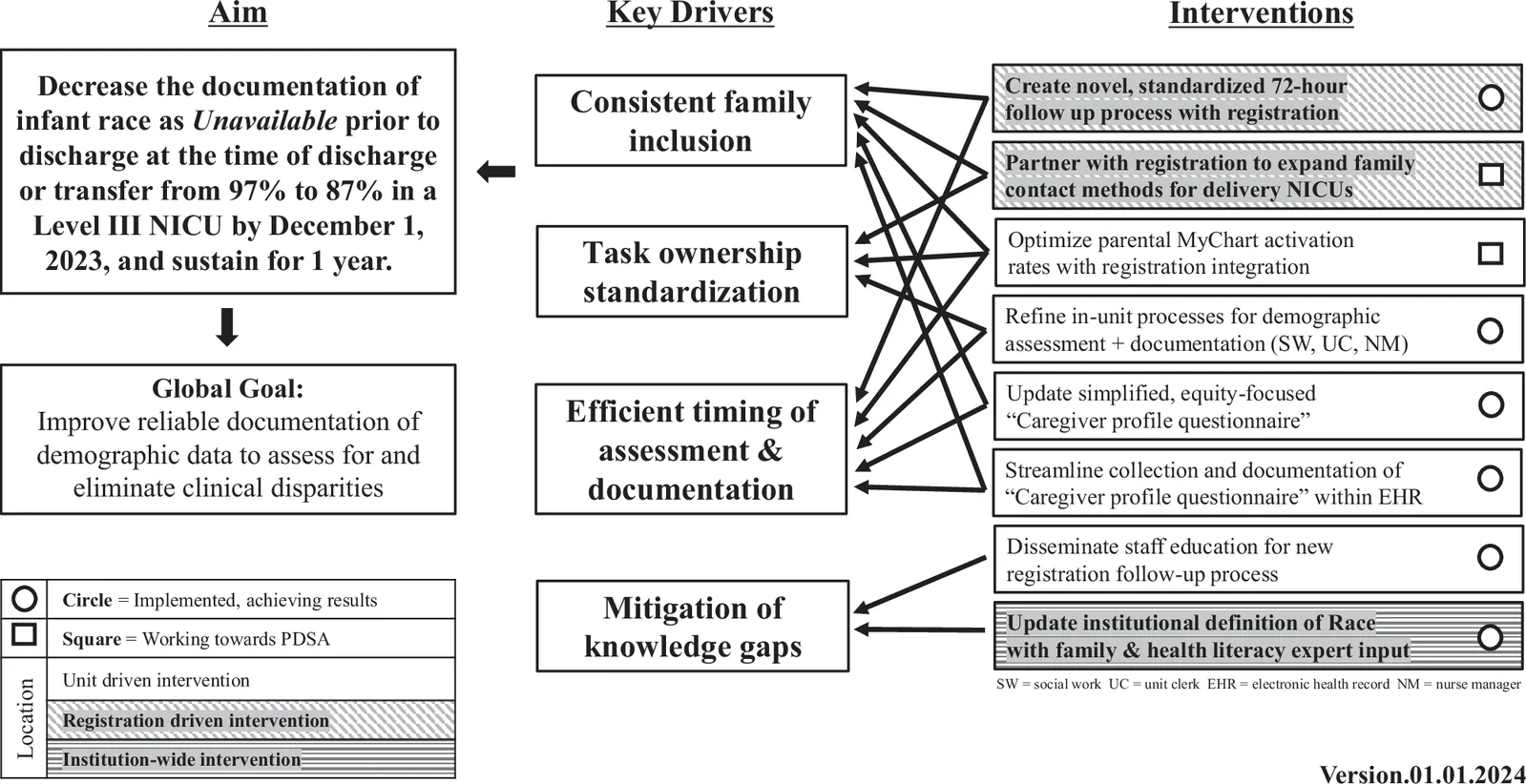
Key Driver Diagram.

Using the Institute for Healthcare Improvement (IHI) model for improvement, we built a multidisciplinary team comprised of nurses, social workers, physicians, unit leadership, and unit-based Parent Advisors, prior NICU parents employed by the hospital to help support families in their NICU journey. The demographic collection process had many touch points, making collaborations with the Registration office, Nursing Informatics, Clinical Informatics, Health Literacy, Family Advisory Committee, and Office of Health Equity. Creation of a Key Driver Diagram with definitions of the global goal and SMART aim identified overlapping needs in the NICU setting and the institution at large (Fig. 2). We used an Impact-Effort matrix to evaluate which Plan-Do-Study-Act (PDSA) cycles would be best for implementation.

At the onset of the project, the institutional definitions of race and ethnicity were collaboratively evaluated and updated, ensuring proper alignment with the 2023 US Department of Health and Human Services recommendations [20]. The institutional racial categories included the Office of Management and Budget federal standards with Hispanic/Latino as a racial option and expanded ethnicity categories (104) to capture the intricacies of our local population.

### Interventions

#### 1. Integrate social work

In October 2023, the first PDSA cycle integrated additional demographic questions into the social work consultation. This intervention fit well given that most families were seen by social work within the first 24 hours of admission. The social workers provided care coordination and support while getting to know the family and their potential needs through a variety of questions, including the names of the parents, contact information, and language preferences. During this conversation, the social workers began providing families with a document co-created with the Office of Health Equity and Health Literacy colleagues to list institutional race and ethnicity definitions and categories. Parents would then self-report the race and ethnicity that their infant best identified with, followed by social work’s EHR documentation.

#### 2. Registration staff call families

In response to identifying the procedure for patient registration failed to engage families or involve interaction with the registration staff who have jurisdiction for all those cared for within our institutional sites, in December of 2023, we developed and implemented a novel follow-up process in which registration staff called families at 72 h following NICU admission if race was still listed as “Unavailable.” This timeframe was intentionally chosen with input from the Family Advisory Committee and the Parent Advisors, to allow time to adjust and settle with the frequently initial high acuity and stress of a NICU and concomitant birthing-parent hospital admission. If a newly admitted NICU patient had infant race documented as “Unavailable,” the chart automatically populated in a working phone call list within the registration office at the main children’s hospital. The staff then called the patient’s caregiver to complete the full registration process, followed by appropriate EHR documentation.

#### 3. Update caregiver profile questionnaire

We collaborated with nursing leadership to integrate additional equity-focused changes to the pre-existing caregiver profile questionnaire: advancing language preference to the first question on the document, adding demographic questions such as infant and caregivers’ race and ethnicity with institutional options included, and a description of why we were asking these questions (Supplementary 1). Through discussion with staff on Gemba walks, we learned that while these questionnaires were often completed by caregivers, they were not uniformly collected or entered into the EHR. Beginning in January 2024, we trialed the updated, equity-focused questionnaire with a targeted documentation approach by staff following caregiver completion. The new process included staff placing completed questionnaires in a central unit location for social workers, patient care assistants, and unit clerks to review and transcribe the demographics in the EHR and file the document in the patients’ paper chart to be scanned into the EHR after discharge. To ensure all staff were aware of the new process, we posted flyers in lounges and off-unit work areas with education given at the staff huddle meetings over a 2-week period.

##### Measures & Study of the Interventions

Our primary outcome measure was the proportion of patients with infant race documented as “Unavailable” for all patients discharged from or transferred out of the pilot NICU site. Processes measures included department responsible for the edit (i.e., social work, registration, family through the EHR patient portal, MyChart, unit staff including RN, unit coordinator, discharge coordinator, provider, unit leadership, or other). This data was collected on infants discharged or transferred via automated weekly EHR data reports, assessing admission, edit, discharge, and transfer timing. There were no readmissions at this off-site, delivery hospital NICU. Data collected originated from a single encounter, occurring in their initial hospitalization. Personnel role was assessed by verifying with information listed on institutional Outlook contact card or verifying with unit leadership. Balancing measures included qualitative and quantitative assessments. Qualitatively, we explore whether time spent by staff was viewed as burdensome and if family interactions were perceived as negative due to the discussion of demographic information. Quantitatively, we tracked the “Decline to answer” response rate. Qualitative data was collected via monthly communication with the team leaders and problem-solving discussions ad hoc. The “Decline to answer” response rate was collected and assessed through the automated weekly EHR data reports.

##### Analysis

Baseline project data was collected January 2022 through April 2023. The project began in May 2023, followed by the first PDSA in October 2023. Staff received weekly feedback with outcome and process measure data plotted over time. All Statistical Process Control (SPC) charts were created using QI Macros SPC Software version 2020.10, which integrates a built-in application for established special cause variation rules based on The Health Care Data Guide [21]. Weekly monitoring and evaluation were performed to assess variations across all measures, to assess contributing contextual elements such as new staff onboarding, staff performance outliers, and reduced administrative availability due to holiday scheduling. Real-time Pareto charting was used to determine ongoing common failure modes, such as length of stay or weekend discharges. While there were multiple clinically oriented QI projects ongoing throughout the Network at the time, there were no significant system changes nor concurrent process improvement projects related to registration or demographic collection throughout the duration of the project. Since this project had begun, the Office of Management and Budget (OMB) federal standards for race and ethnicity collection were updated, combining race and ethnicity into one question, encouraging the selection of multiple demographic categories as it applies, and adding of a new category, Middle Eastern or North African, as of March 2024 [22]. Due to the timing of this project and the required complexity to change the EHR, institutional standards were not updated to match the new recommendations. There are current efforts underway to create an implementation plan to incorporate these recommendations in the future.

##### Ethical considerations

The project was determined to be Quality Improvement without inclusion of human subjects’ research and was therefore exempt from our Institutional Review Board per policy. All methods were performed in accordance with guidelines and regulations with the Standards for Quality Improvement Reporting Excellence Guidelines were followed [23]. The authors have no conflict of interest to this work.

## Results

**Fig. 3:**
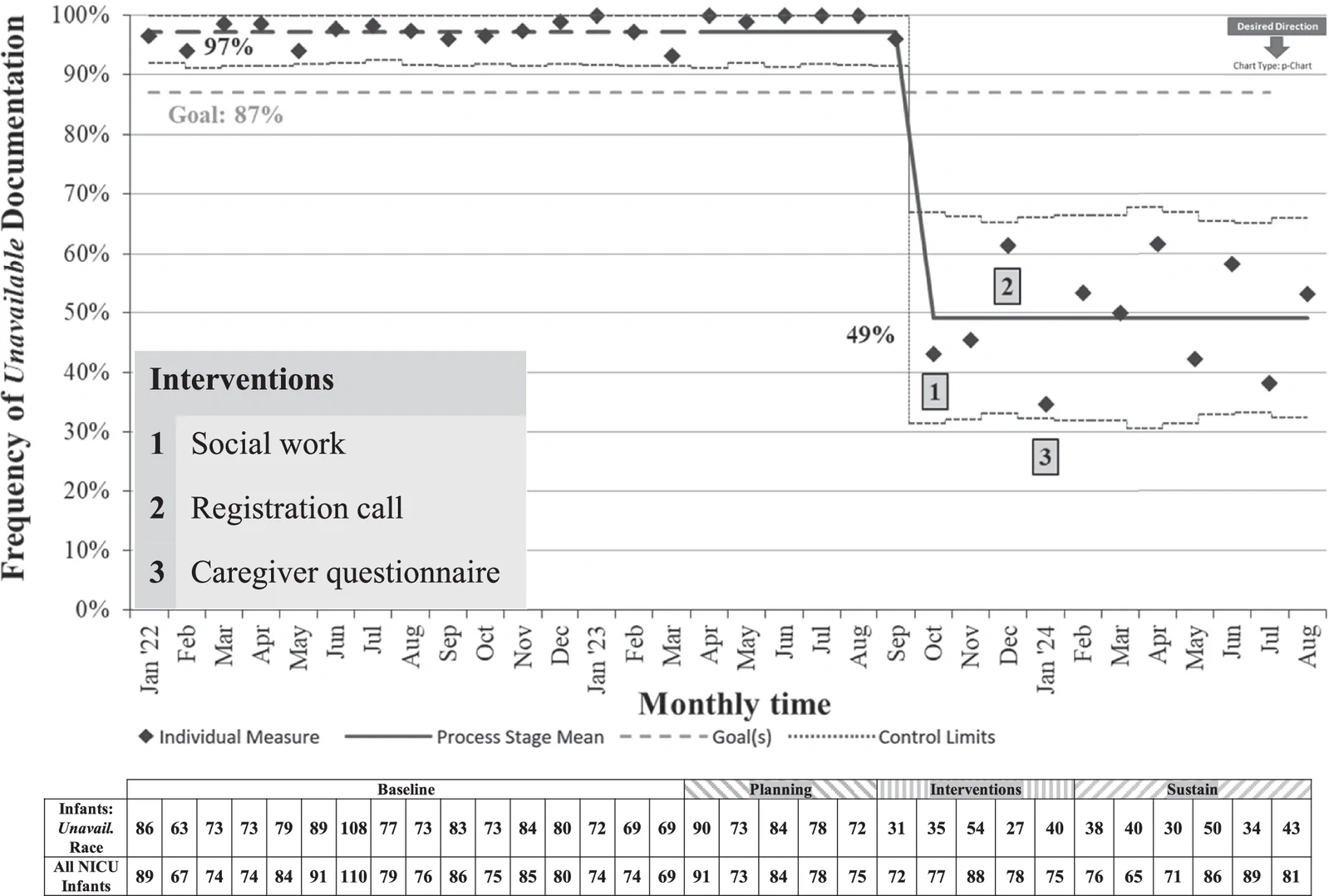
P-chart of Infant Race Documented as *Unavailable*.

The 16-month baseline period and 5-month planning period had an average of 97% “Unavailable” documentation (Fig. 3). The SPC chart shows a centerline shift after observing special cause variation (Rule 1) beginning October 2023, with a decrease from 97% to 49%, which coincided with implementation of the first intervention, social work integration [21]. There were no other observed signals of special cause variation. We surpassed our original goal to reduce “Unavailable” documentation by 10% during the study period, which was sustained. Throughout the entire study duration, the average monthly admission rate remained stable between 78 and 80 infants, with 2546 infants included in total.

**Fig. 4:**
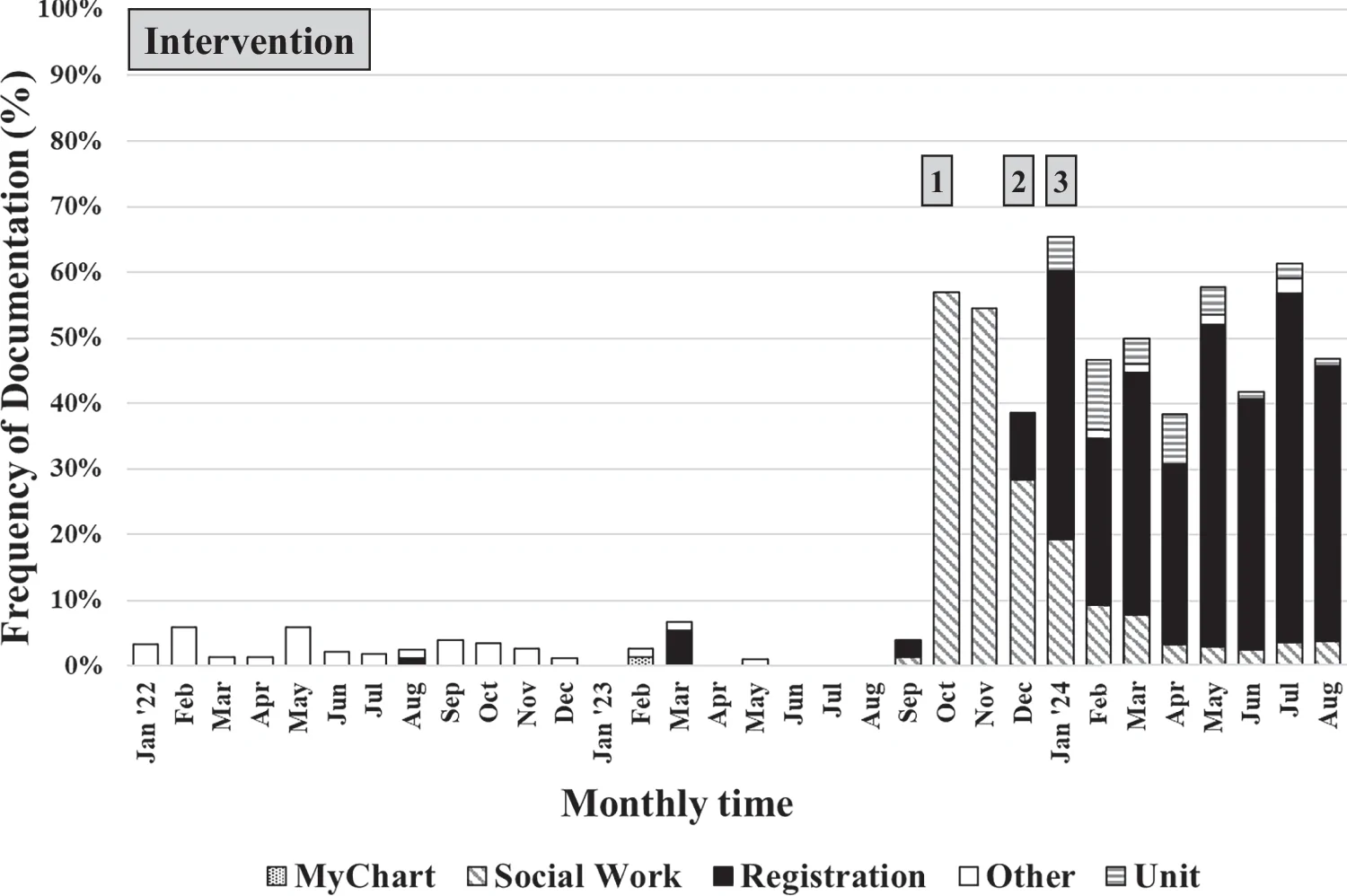
Caregiver-reported infant race by personnel role. Process measure over time.

Staff documenting infant race was tracked throughout the study as a process measure, with caregiver-reported infant race minimally documented during the baseline and planning periods (Fig. 4). In alignment with the first intervention, goal documentation performed by social work increased to 57% in October 2023. As registration staff began connecting with families through the second intervention, a gradual increase in documentation by this group occurred, from 10% in December 2023 to 25% in February 2024. A smaller portion of appropriate documentation, 5–12%, was performed steadily by unit staff in conjunction with the third intervention, beginning in January 2024. As registration and unit staff discussed demographic information with caregivers at the time of admission or quickly thereafter, the amount of remaining incomplete documentation fields available for social work to fill gradually decreased. In April 2024, social work began gathering caregiver provided demographic information and sending the information to registration staff, who documented the data due to an issue with demographic data inconsistently carrying across EHR encounters. This change capitalized on the in-person relationship and connection made by in-house social workers, while utilizing skills and training of registration staff to ensure the most complete, well-documented data. Thus, process measures indicate a decrease in social work documentation with a paired increase in registration documentation that fails to capture the interplay of the two job functions.

**Fig. 5:**
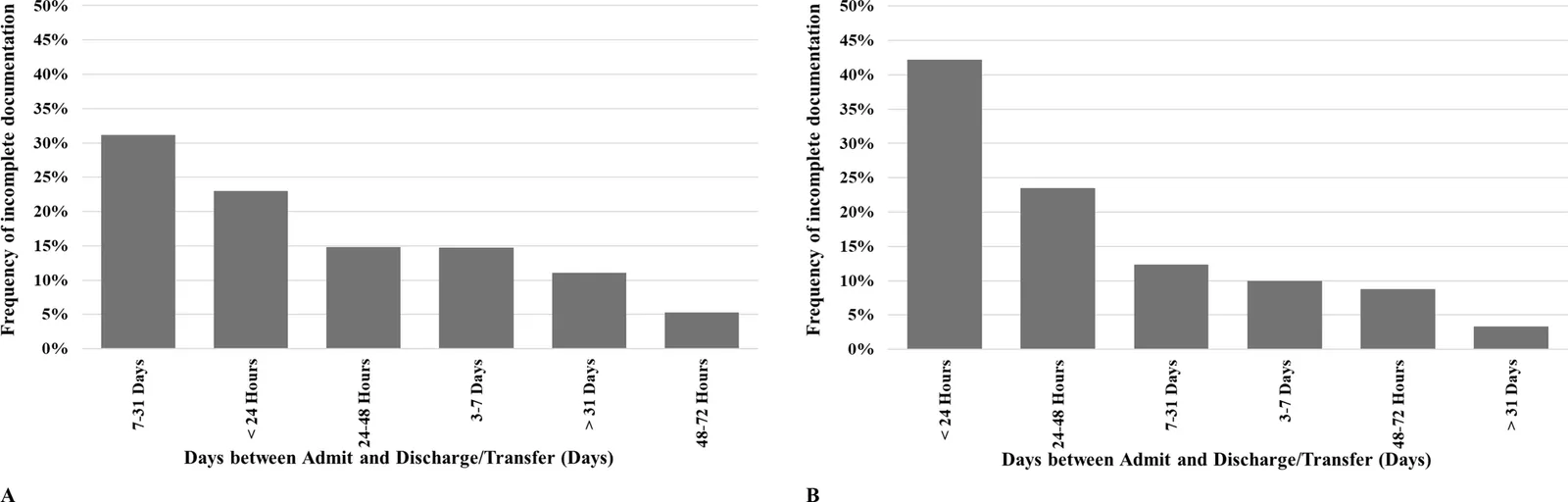
Pareto chart of missed documentation opportunities by length of stay. **A** Baseline period, **B** Intervention period.

Additional process and data analyses have shown that short-stay admissions remain our largest opportunity of improvement (Fig. 5). Other missed populations have been those with access barriers, such as lack of access to stable phone numbers or service and speaking languages other than English. While we observed increased representation in every racial subpopulation, we saw the largest percent improvement in capturing our Asian, Latino, and Multi-racial populations, respectively (Supplementary 2). If we assume the institutional population represents this NICU population, we have the greatest need to improve documentation in the White and Black groups. Given the significant improvement surpassing and sustaining goal documentation for six months, the pilot was expanded early to a second, network-wide study with inclusion of all 10 NICUs. Sustained documentation has persisted for over 1-year but is not shared here as it reflects ongoing expansion work.

Negative system impacts were avoided with stability in the balancing measures tracked. Social work reported less than five minutes required to gather the necessary information and ensure correct EHR documentation. Registration reported each phone call process to last 2–3 min in total, with easy integration into their workflow by utilizing down time throughout the day to complete the calls and document. To date, there have been zero perceived negative interactions with families in conjunction with discussion of demographic information. “Decline to answer” response rates were 0–1% from the baseline through the intervention periods, January 2022–January 2024, with an increase to 1.7% in the sustain period beginning in February 2024.

## Discussion

This study shows that revising the processes of infant demographic collection and documentation is complicated, but feasible, achieving a significant, near 50% reduction in “Unavailable” infant racial documentation at our level III NICU. Core to this success was the utilization of both IHI and Lean Six Sigma practices with multidisciplinary team engagement. Understanding the baseline processes from frontline perspectives prior to action led to two types of interventions: process changes at the local, unit-level, and at the broader, institutional-level.

This project led our journey to assess for and impact clinical disparities as a critical first step of validating the completeness and accuracy of patient demographic information. It serves as a blueprint for improving data collection for applied analyses in both the clinical outcome improvement setting within our hospital system and larger population health settings such as the Ohio Equity Institute. Throughout the project, we clearly communicated the goal of improving our understanding of the population we serve and ensuring we provide the best possible care to all infants. Open discussions with staff and families explaining this mission led to increased buy-in and reduced friction. Safe spaces were held for staff at unit huddles, unit meetings, and within Gemba walks to address concerns and hesitations while providing guidance for future caregiver conversations with standardized language, approaches, and responses for common concerns.

### Interpretation

This project addresses the national and state issues faced with patient or proxy-reported infant demographic data. There are no national standards for reporting infant demographics, even related to birth certificates; despite the wide variation in reporting practices between states, birth certificates are recognized as the most reliable source of population-level demographic data [24]. Many national records include infant demographics that are transcribed from the birthing parent’s self-reported information, directly excluding the non-birthing parent or assuming identical demographic information in their partner, while others include infant demographic reporting determined by staff “personal observation” [24]. Furthermore, inconsistencies and inaccuracies of infant racial and ethnicity data are common and have disparities with the most significant impact on infants of color and those with demographically discordant parents [24]. Within pediatrics, the Pediatric Health Equity Collaborative developed a framework specific with recommendations to include demographic collection for patients and parents or caregivers with an emphasis on self-reporting, as able [25]. A Pediatric Emergency Research Working Group released publication consensus guidelines in 2024, highlighting the inclusion of the demographic collection methodology and use of an institutionally standardized format of self-reported data collection [26]. Recommendations supporting caregiver-reported, high quality demographic data are crucial to advancing pediatric research, echoed by JAMA Pediatrics [27]. Pediatric institutions routinely report de-identified neonatal data to state and national QI and research collaboratives to advance care of the infant population. Organizations such as Ohio Equity Institute (OEI), Solutions for Patient Safety (SPS), the Vermont Oxford Network (VON), the Children’s Hospital Neonatal Consortium (CHNC), and the Center for Disease Control (CDC) depend on the accuracy of this data to inform focus areas, underscoring the need to understand internal data collection processes and ensure data reliability. Importantly, VON and CHNC have used maternal race as a proxy for infant race, CDC and OEI use Vital Statistics data from birth certificates, and SPS uses patients’ demographics from the EHR; all of which raise concerns as to the appropriateness and validity of that information. For example, all families at our institution complete a state-issued birth certificate during hospitalization. Yet, there are no current workflows to transfer this information into the EHR, making it impossible to utilize this information. The categorical options on the state-issued birth certificates are also much narrower compared to the expanded institutional options that allowed for more specific ethnic identification. These concerns held high risk for missing important details with a reduced opportunity for highly accurate information, leading us to innovate with novel collection and documentation methods with superior fit for our setting and population needs.

The lack of standardization is further amplified when we consider the multi-healthcare system fusions that occur with neonatal care delivery models throughout the country. The NICU registration process for demographic collection often serves as the first interaction with the pediatric healthcare system. A disorganized approach can cause potential negative impacts and first impressions for families. This disjointed process was observed during this study, occurring at a delivery, “off-site” NICU affiliated with a larger children’s hospital. While some of the resources and staff availability may be different at this “off-site” location, we use a unified physician group and set of protocols to ensure high-quality, standardized care delivery throughout the NICU network, including at delivery sites that offer high-quality obstetrical care to the dyad in locations closer to families’ homes. Throughout the project, it became increasingly apparent we needed the same standardized process for other care aspects, including registration.

Through our Gemba walks, we saw that caregivers at the delivery center NICU were never in contact with the main campus registration staff, and thus, were never asked to self-report their infant’s demographic information for intake into the EHR. Prior attempts to address this issue of having demographic questions asked by the bedside nurse at discharge failed due to adding yet another task to an already busy workload, scope of practice concerns, and timing difficulties with a “getting to know you” clerical task being put off until the end of a potentially long NICU journey. The first intervention of incorporating registration questions into the social work consultation helped to ensure caregiver self-report and proved to be the most significant overall. A similar focus on staff training and standardization of the registration process yielded successful improvements for the University of Texas’ efforts in adult patient demographic collection over a 5-year period [28]. One key difference between the two studies is the setting. While this may be streamlined in settings with on-site registration staff, our study had separated locations between the registration staff and the NICU with the caregivers and the infants. We found that by training a smaller, core group of unit staff, we were able to mitigate this common issue within NICU settings and have a smaller impact on the larger system. The improvement was then sustained through the novel, direct communication process between registration staff and caregivers within the second intervention. This process was the most complex, involving a larger number of staff to train with new barriers related to phone calls, including technology requirements, optimal timing, and interpreter needs. Despite these challenges, this intervention had a positive impact. The effectiveness of large, systems-based interventions, with specific importance to staff training, EHR incorporation, and leadership buy-in, was highlighted in an adult healthcare database systematic review [13].

We also found it crucial to continue weekly, micro-assessments of ongoing measures. Through our weekly report reviews it was recognized that the balancing measure of “Declined to answer*”* responses had increased following the phone call intervention. Upon further review, one employee was identified as the documenter for all 8 of these responses. Following direct education and engagement with this employee, appropriate collection and documentation improved. We also found dips in data during holiday times with restricted staff hours for social work and registration, prompting us to brainstorm future interventions of direct patient engagement (i.e., MyChart, registration tablets or kiosks, etc.).

When comparing our study to similar efforts to improve complete and accurate demographics of infants specifically, there is a paucity of comparisons. Boston Children’s Hospital recently found a high amount of missing demographic data among children admitted through a transfer process to intensive care units, with recognition of a differing process from those admitted through the emergency department [22]. Following standardization of the demographic collection processes through staff role delineation and EHR integration, they saw a reduction in missing demographic data [29]. While their study included infants in the NICU, their level IV setting as a tertiary referral center had children’s hospital registration staff at the front desk of the unit, making this example less generalizable to “off-site”, delivery NICU settings such as our pilot site.

### Limitations

While it was crucial for the project to document within the gold-standard source for the infant’s health information created at birth, with likelihood to persist with them through their childhood as they engage in pediatric care, working within the EHR proved challenging. To help minimize the significance of these anticipated barriers, we involved Nursing Informatics and a Clinical Informatics physician colleague within the multidisciplinary team. Wide editing access for all staff creates difficulty with ensuring data validity. A significant amount of staff education was performed in an institutionally standardized format to help overcome this. Limited ability to customize the demographic documentation categories and question format proves difficult with the updated OMB recommendations [22].

Additional staff education and training were provided on the goal of the project, with common definitions and concerns discussed, but it was ultimately up to individual staff members to lead culturally sensitive conversations, ensure the data is self-reported from the caregiver, and document appropriately [22]. While there were no reported negative caregiver interactions, we did not elicit standardized caregiver evaluations on their registration experience.

Despite surpassing our goal with a 48% reduction in “Unavailable” infant racial demographic documentation, our ultimate goal is to work toward 100% caregiver-reported demographic data. We continue to perform ongoing process and data analyses to identify the largest opportunities for further improvement, such as populations admitted for short-stays and those with access barriers. Similar to the strategies modeled by the Ohio Equity Institute and in published equity-focused QI standards, identified priority issues as we continue ongoing improvement efforts across the expanded, network-wide initiative [3, 5].

## Conclusions

This pilot project at an off-site, delivery hospital NICU showed a 48% reduction in infant demographic data documented as “Unavailable” and sustained for a 6-month period. With collaborative, conversational engagement and both unit and system-wide interventions, we have greatly shifted the culture to prioritize caregiver-reported infant demographic data collection in a high-acuity NICU setting. Our results demonstrate that by applying these pediatric specific recommendations to the complex environment of a NICU, substantial improvement in accurate demographic data with sustainability is possible. This study’s success fueled an expansion to our 10-unit Neonatal Network, with promising positive momentum. We believe that this systematic work can be adapted by other institutions and is an imperative prequel to improving infant health disparities, as called for by our state and other population health experts nationally.

## Supplementary information

Questionnaire introduction

Improvements in Capturing Demographic Subpopulations

## Data Availability

The datasets generated during and analyzed during the current study are not publicly available due to containing protected health information, but are available from the corresponding author on reasonable request.
