# Supplementary material for: Sustained improvement in caregiver-reported infant demographic collection: a pilot towards equitable outcomes in the neonatal intensive care unit
Source: J Perinatol. 2026 May 18;46(10):1979–85. doi: 10.1038/s41372-026-02676-z (PMC13680261; doi:10.1038/s41372-026-02676-z)
Supplement: Supplementary file 1 [file 41372_2026_2676_MOESM1_ESM.pdf]

NICU Admission Questionnaire

Welcome to the NICU and Congratulations. Giving the best care to your baby starts with us getting to know your family and how we can best serve you. We are committed to providing high-quality, equitable healthcare for all families. This information allows us to track our health outcomes.

Please see Race and Ethnicity definitions on the back of this paper.
