# Supplementary material for: Sustained improvement in caregiver-reported infant demographic collection: a pilot towards equitable outcomes in the neonatal intensive care unit
Source: J Perinatol. 2026 May 18;46(10):1979–85. doi: 10.1038/s41372-026-02676-z (PMC13680261; doi:10.1038/s41372-026-02676-z)
Supplement: Supplementary file 2 [file 41372_2026_2676_MOESM2_ESM.pdf]

S2. Improvements in Capturing Demographic Subpopulations

	Asian	%	% impv	Black	%	% impv	Latino	%	% impv	Multi-racial	%	% impv	White	%	% impv	Other	%	% impv	Total
Baseline	2	**0.15%**		55	**4.26%**		13	**1.01%**		19	**1.47%**		125	**9.67%**		2	**0.15%**		1292
Study	4	**1.01%**	**649.25%**	45	**11.31%**	**265.60%**	22	**5.53%**	**549.36%**	25	**6.28%**	**427.14%**	140	**35.18%**	**363.58%**	1	**0.25%**	**162.31%**	398
Sustain	6	**1.30%**	**840.78%**	39	**8.46%**	**198.73%**	15	**3.25%**	**323.38%**	31	**6.72%**	**457.27%**	164	**35.57%**	**367.70%**	0	**0.00%**	**0.00%**	461
Institutional	28776	**4.43%**		168517	**25.92%**		51123	**7.86%**		45179	**6.95%**		346744	**53.33%**		1988	**0.31%**		650137
Institutional comparison		**3.12%**			**14.61%**			**2.34%**			**0.22%**			**17.76%**			**0.05%**		
